# A trend analysis of the burden of lower extremity peripheral arterial disease in China, 1990 to 2021: based on the Global Burden of Disease Study 2021

**DOI:** 10.3389/fpubh.2025.1506748

**Published:** 2025-06-19

**Authors:** Chen Zhang, Mo Chen, Xintao Huang, Hao Lu, Junyu Wang, Enze Liu, Landan Xiao, Huisheng Deng

**Affiliations:** Department of General Practice, The First Affiliated Hospital of Chongqing Medical University, Chongqing, China

**Keywords:** GBD, PAD, China, burden of disease, AMIRA

## Abstract

The present study provides a thorough trend analysis of the burden of lower extremity peripheral arterial disease (PAD) in China during 1990–2021, based on data from the Global Burden of Disease Study 2021. Lower extremity PAD is an atherosclerotic disease that causes obstruction of blood vessels supplying the legs, presenting as intermittent claudication, rest pain, non - healing wounds, ulcers, or gangrene, and may lead to limb amputation or death due to critical limb ischemia. Our analysis covers prevalence, incidence, mortality, years lived with disability (YLDs), years of life lost (YLLs), and disability - adjusted life years (DALYs). A key finding of this study is from the Age - period - cohort (APC) analysis. It shows that age and period effects are risk factors for the incidence and mortality of PAD, while birth cohort effects have a protective role. Additionally, projections using the Autoregressive Integrated Moving Average (ARIMA) model indicate that the risk of death from PAD will increase for males in the future. Through Joinpoint regression analysis, we delineate the temporal trends. Considering China’s aging population, the growing disease burden from economic progress, and the rapidly changing healthcare landscape, these findings highlight the escalating challenge of PAD. The study’s predictions serve as a warning of the continued rise in PAD incidence and emphasize the urgent need for public health interventions to address the increasing burden.

## 1 Introduction

Lower extremity peripheral artery disease (PAD) is defined by atherosclerotic obstruction of the arteries supplying the legs, affecting approximately 230 million people worldwide (1). According to the “China Cardiovascular Health and Disease Report,” the current number of people with cardiovascular diseases in China is 330 million, including 13 million with stroke and 11.39 million with coronary heart disease, among which the prevalence of peripheral arterial disease of the lower extremities is as high as 6.6% (2). PAD can manifest as pain in one or more lower limb muscle groups during physical activity, namely intermittent claudication, atypical pain, pain at rest, or non-healing wounds, ulcers, or gangrene, with severe limb ischemia potentially leading to amputation or even death of the patient (3).

Asia, particularly China, is facing an increasing challenge from lower extremity peripheral artery disease (PAD). It is estimated that the Asian population accounts for approximately 60% of the global population, while China’s population represents 18%, forming a large base of disease burden. Despite differences in economic conditions and healthcare systems across the world, the impact of PAD on human health is widespread and severe (4). Particularly since the beginning of the 21st century, while the incidence of PAD in Western countries has been stabilizing, the incidence in emerging industrialized nations of South America, Eastern Europe, Asia, and Africa has been rising rapidly (5). As one of the world’s largest lower-middle-income countries, China has experienced rapid economic development over the past few decades and a significant increase in the burden of non-communicable diseases. It is estimated that from 2000 to 2020, the total number of PAD patients in China increased by 40% (6). The burden of PAD has become a challenge for China’s healthcare system, similar to Western nations (7).

However, there is a relative scarcity of epidemiological studies on lower extremity PAD in China at present, limiting researchers’ in-depth understanding of PAD epidemiological trends. This study is based on the latest data from the Global Burden of Disease (GBD) 2021 and aims to reveal the long-term trends in the disease burden of lower extremity PAD in China over the past 30 years, including prevalence, incidence, mortality, Years Lived with Disability (YLD), Years of Life Lost (YLL), and Disability-Adjusted Life Years (DALY). Furthermore, this study employs the Autoregressive Integrated Moving Average (ARIMA) model (8, 9) to forecast the trend of PAD disease burden in China for the next 15 years, in hopes of providing a scientific basis for the prevention and control of PAD.

## 2 Methods

### 2.1 Overview

The 2021 Global Burden of Disease (GBD) report provides us with a macro perspective, covering 371 types of diseases and injuries across 204 countries and regions. The report employs numerous statistical methods to provide detailed estimates of prevalence, incidence, deaths, and health years of life lost (YLL) and years lived with disability (YLD), and calculates disability-adjusted life years (DALY) (Equation 1) as a comprehensive metric for measuring the burden of disease. These estimates are categorized by cause, age, sex, year, and geographic location, providing invaluable data support for research and policy-making in the field of global health (9).


(1) DALYs=YLLs+YLDs

The original data for the GBD-estimated incidence and mortality rates of lower extremity PAD in China primarily come from the Chinese Center for Disease Control and Prevention (CDC) mortality reporting system, Disease Surveillance Points (DSP), and Maternal and Child Health Surveillance System. These data are widely representative due to their nationwide coverage.

The dataset utilized in this study encompasses the burden data of lower extremity PAD in China from 1990 to 2021, including age-standardized rates (ASRs) (Equation 2), age-specific rates, and 95% uncertainty intervals. All data have undergone rigorous quality control, and detailed information is available on the official website of the Global Burden of Disease project.^1^


(2) $$\mathrm{ASR}=\frac{{\sum^{A}}_{i=1}\alpha_{i}w_{i}}{{\sum^{A}}_{i=1}w_{i}}$$

The variable *α_i_* denotes the age-specific rate, while *w_i_* signifies the weight derived from the standard population corresponding to each of the respective age groups. Additionally, A represents the total number of age groups.

Lower extremity PAD is caused by plaque buildup in the peripheral vessels. The gold standard definition is having an ankle-brachial index (ABI) of less than 0.90, with leg pain on exertion called intermittent claudication in those with an ABI below that threshold.

This study has been approved by the Institutional Review Board of the First Affiliated Hospital of Chongqing Medical University. As the research is based on publicly available data, no special permission is required.

### 2.2 Joinpoint regression analysis

The Joinpoint regression model is a set of linear statistical models (10) used to assess the trends in disease burden caused by lower extremity PAD over time. The model uses the least squares method to estimate the patterns of disease rate changes, effectively avoiding the subjectivity that traditional trend analysis based on linear trends may bring. This method calculates the sum of squares of the residuals between the estimated and actual values, allowing the model to identify significant change points.

We used Joinpoint software (version 4.9.1.0, developed by the National Cancer Institute in Rockville, Maryland, USA) to construct this model. Each *p*-value is obtained using the Monte Carlo method, and the overall asymptotic significance level is maintained through Bonferroni correction. The Average annual percentage change (AAPC) (Equation 3) is calculated as the geometric weighted average of various annual percentage change values in the regression analysis. Typically, a *p*-value less than 0.05 is considered statistically significant.


(3) $$\text{AAPC}=(e^{\beta 1}-1)\times 100\%$$

AAPC is typically calculated using a log-linear regression model. Firstly, time series data (such as incidence or mortality rates) are log-transformed, and time is used as the independent variable in the regression to obtain the regression coefficient (β1).

### 2.3 Age-period-cohort (APC) analysis

Over the past 80 years, the APC model has been widely applied in the fields of sociology and epidemiology. The APC model, based on the Poisson distribution, can reflect the temporal trends of diseases by age, period, and cohort, as well as after adjusting for age, period, and cohort. However, the perfect collinearity between age, period, and cohort makes it difficult to estimate a unique set of influences for each, thus, the problem of non-identifiability may still persist. We employ an APC model analysis technique explained by Carstensen (11) from the Lexis diagram, to visually express the impact of age groups, period intervals, and birth cohort intervals on incidence and mortality rates. We then use the Intrinsic Estimator (IE) method (12) to visually express the impact of individual factors on incidence and mortality rates. The Intrinsic Estimator method resolves the collinearity issue through zero constraints. We inquired about the incidence and mortality rates for every 5-year age group from 1990 to 2021 according to the GBD, as well as the annual population estimates. The GBD groups those under 5 years old and over 95 years old into one category. For APC model fitting, age groups are defined as 0–4, 5–9, 10–14.0.95–100. In the graph, 0 represents the group under 5 years old. The 5-year periods are represented as (1992–1996, 1997–2001.0.2017–2021).

The APC_IE model is analyzed using Stata/MP software (version 15.0, StataCorp, College Station, TX, USA). Additionally, R software (version 4.3.3, R Foundation, Vienna, Austria) with the ‘Epi’ and ‘ggplot2’ packages is utilized for visualization.

### 2.4 Autoregressive integrated moving average (AMIRA) predictive analysis

The ARIMA model can be used for predictive analysis to estimate the burden of disease (8). The ARIMA model adopts the form ARIMA (p, d, q) × (P, D, Q). Parameters d (the degree of differencing) and D (the moving average) are the numbers of differences required for stabilizing the time series. Parameters p (the order of autoregression) and q (the order of the moving average) are simple numeric parameters. Parameters P (seasonal autoregression) and Q (seasonal differencing) are seasonal parameters, and s is the length of the seasonal cycle. The detailed model construction process and explanation have been elaborated in previous studies (13). We used the auto.arima function in the ‘forecast’ R package to automatically fit the optimal ARIMA model. The R package is publicly available on GitHub,^2^ and then the ‘ggplot2’ R package is used for visualization.

The analysis is conducted using R software (version 4.3.3, R Foundation, Vienna, Austria).

## 3 Results

### 3.1 Descriptive analysis

**Figure 1:**
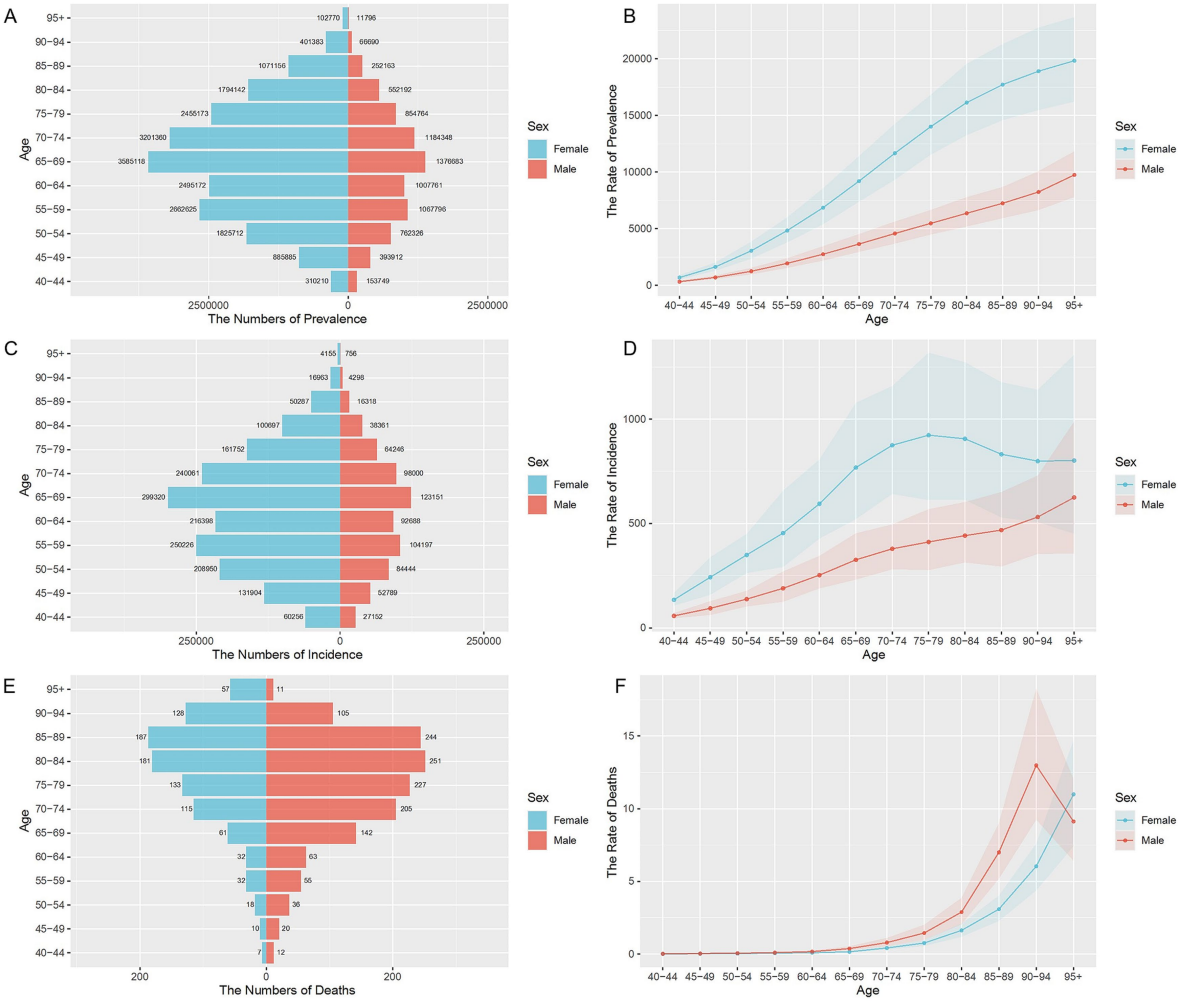
Age-specific numbers and age-standardized prevalence, incidence, and mortality rates of PAD in China, 2021. **(A)** Age-specific prevalence number. **(B)** Age-standardized prevalence rate. **(C)** Age-specific incidence number. **(D)** Age-standardized incidence rate. **(E)** Age-specific mortality number. **(F)** Age-standardized mortality rate.

In China, in 2021, there were 2,447,372 (95% CI: 2,109,991 to 2,854,291) new cases and 2,332 (95% CI: 1,808 to 2,954) deaths attributed to lower extremity PAD. The age-standardized rates for lower extremity PAD in 2021, including age-standardized prevalence (ASPR), incidence (ASIR), mortality (ASMR), DALYs, YLDs, and YLLs, were 1331.13 cases (95% CI: 1147.49 to 1544.17) per 100,000, 112.66 new cases (95% CI: 97.75 to 130.73) per 100,000, 0.13 deaths (95% CI: 0.10 to 0.16) per 100,000, 8.36 DALYs (95% CI: 4.87 to 14.33) per 100,000, 6.35 YLDs (95% CI: 3.01 to 12.61) per 100,000, 2.01 YLLs (95% CI, 1.54 to 2.54) per 100,000. The specific results are shown in Table 1, which lists the disease burden by gender. As shown in Figure 1, it displays the number and age-standardized rates of prevalence, incidence, and mortality for different age groups of lower extremity PAD in 2019 (A, C, E for numbers; B, D, F for rates). In terms of prevalence, the rate shows a gradually increasing trend with age. Among them, the number and rate of disease in women are consistently higher than in men, at all ages. In terms of incidence, it shows an increasing trend with age. Among them, the incidence rate in women peaks between 70 to 79 years old and then shows a decreasing trend, but it remains higher than in men. Surprisingly, in terms of mortality, the male death rate increases progressively with age between 40 to 95 years and remains consistently higher than in women. Until after the age of 95, the male death rate drops significantly, and a phenomenon where the female death rate exceeds that of males for the first time occurs. The age-standardization rates of DALYs, YLDs, and YLLs show similar trends across genders and age groups (see Supplementary Figure 1).

**Table 1:** All-age cases and age-standardized prevalence, incidence, deaths, YLLs, YLDs, and DALYs rates in 2021 for PAD in China.

Measure	Num	All-ages cases		Rate	Age-standardized rates per 100,000 people	
	Both	Female	Male	Both	Female	Male
Prevalence	28,474,886 (24,446,553,33,220,180)	20,790,704 (17,849,890,24,184,371)	7,684,182 (6,524,263,9,027,019)	1331.13 (1147.49,1544.17)	1854.94 (1596.94,2148.61)	747.8 (641.62,868.51)
Incidence	2,447,372 (2,109,991,2,854,291)	1,740,972 (1,500,126,2,028,607)	706,400 (607,066,830,957)	112.66 (97.75,130.73)	155.62 (134.88,180.26)	67.49 (58.71,78.53)
Deaths	2,332 (1808,2,954)	961 (717,1,217)	1,371 (963,1851)	0.13 (0.1,0.16)	0.1 (0.07,0.12)	0.18 (0.13,0.24)
DALYs	171,757 (99,158,301,528)	114,748 (60,714,215,456)	57,009 (37,840,86,959)	8.36 (4.87,14.33)	10.35 (5.52,19.06)	6.06 (4.14,9.1)
YLDs	132,593 (63,047,268,917)	99,752 (47,293,201,929)	32,840 (15,472,65,398)	6.35 (3.01,12.61)	8.92 (4.23,17.78)	3.31 (1.56,6.48)
YLLs	39,164 (29,897,49,968)	14,996 (11,143,19,138)	24,168 (16,880,33,045)	2.01 (1.54,2.54)	1.42 (1.06,1.82)	2.75 (1.93,3.72)

DALYs, Disability-Adjusted Life Years; YLDs, Years Lived with Disability; YLLs, Years of Life Lost.

**Figure 2:**
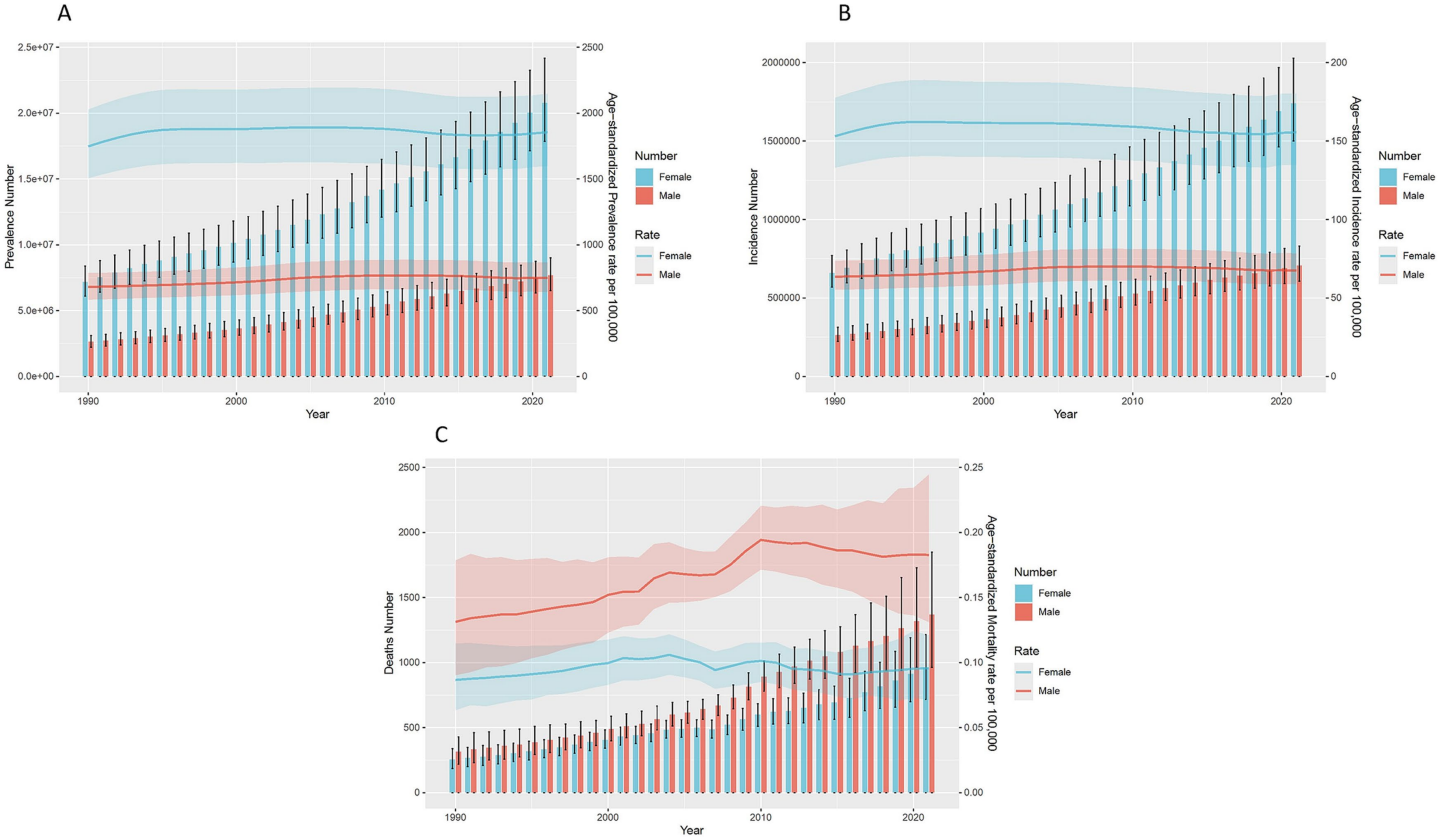
Trends in the all-age cases and age-standardized incidence, mortality, and DALYs rates of IBD by sex from 1990 to 2021. **(A)** Prevalence number and rate. **(B)** Incidence number and rate. **(C)** Mortality number and rate.

From 1990 to 2021, the age-standardized prevalence rate (ASPR) per 100,000 people increased from 1250.85 to 1331.13. The age-standardized incidence rate (ASIR) per 100,000 people increased from 109.57 to 112.66. The age-standardized mortality rate (ASMR) per 100,000 people increased from 0.1 to 0.13. Concurrently, age-standardized DALYs, YLDs, and YLLs all showed varying degrees of increase. For details, see Supplementary Table 1. Figure 2 illustrates the trends in prevalence (A), incidence (B), mortality (C), and the number of cases. With the increase in years, the growth in incidence and prevalence rates has shown a slow trend. However, the number of cases has shown a rapidly increasing trend, especially among females. While the mortality rate generally fluctuates with calendar years, male mortality rates reached their peak around 2010 and then showed a declining trend, whereas female mortality rates reached their peak around 2004 and then showed a declining trend. Regardless of mortality rates or the number of deaths, males consistently had higher rates than females over the 30-year period. The trends in DALYs, YLDs, and YLLs over the years are similar; for details, see Supplementary Figure 2.

### 3.2 Joinpoint regression analysis

**Figure 3:**
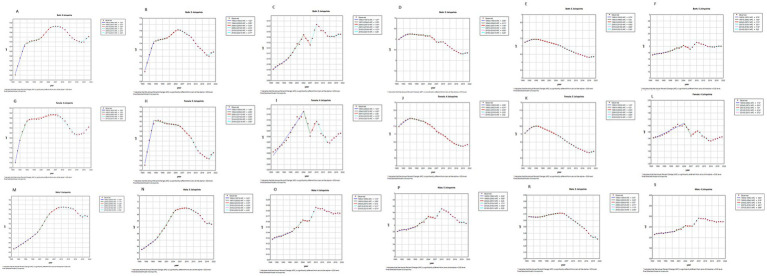
Joinpoint regression analysis of the sex-specific age-standardized incidence rate for PAD in China from 1990 to 2021.

Figure 3 illustrates the APC and AAPC changes in the disease burden of lower extremity PAD in China from 1990 to 2021. In terms of ASPR (A), the results indicate a significant upward trend from 1990 to 1994, followed by a moderation in the upward trend from 1994 to 2009. A downward trend from 2009 to 2017 was followed by an upturn from 2017 to 2021, with an overall increasing trend. The overall trend is upward with an AAPC of 0.19 (95% CI: 0.16–0.23). Among them, males (M) showed the largest increase (AAPC = 0.32, 95% CI: 0.27–0.36), but experienced a downward trend from 2009 to 2021. For females (G), aside from a slow downward trend from 2008 to 2016 (APC = −0.45, 95% CI: −0.50 to −0.39), an upward trend was observed in other years.

In terms of ASIR (B), the results show a similar increasing trend from 1990 to 2005 in both sexes as observed with ASPR. There was a decreasing trend from 2005 to 2018, followed by a slight rebound from 2018 to 2021. Overall, there is a modest upward trend (AAPC = 0.08, 95% CI: 0.06–0.11). Among them, males (N) had the largest increase (AAPC = 0.20, 95% CI: 0.17–0.24). Surprisingly, the annual average rate of change is 2.5 times that of the average for both sexes and 4 times that of females (H).

In terms of ASMR (C), the results show significant fluctuations from 1990 to 2021, but an overall increasing trend with an AAPC of 0.73 (95% CI: 0.50–0.96). Among them, males (N) had the largest increase with an AAPC of 1.00 (95% CI: 0.70–1.30), especially from 2007 to 2010, with an APC of 5.11 (95% CI: 3.18–7.08). In terms of DALYs (D, J, P), the results show a decreasing trend in both sexes with an AAPC of −0.24 (95% CI: −0.30 to −0.18). However, an increasing trend is observed in males with an AAPC of 0.13 (95% CI: 0.01–0.26).

In terms of YLDs (E, K, R), the results show a decreasing trend from 1990 to 2021 in both sexes with an AAPC of −0.43 (95% CI: −0.46 to −0.40). In terms of YLLs (F, L, S), the results show an increasing trend in both sexes with an AAPC of 0.47 (95% CI: 0.30–0.64). Among them, the annual average rate of change for males is more than 14 times that of females. Detailed data are presented in the Supplementary Table 2.

### 3.3 The effects of age, period, and cohort on incidence and mortality rates

**Figure 4:**
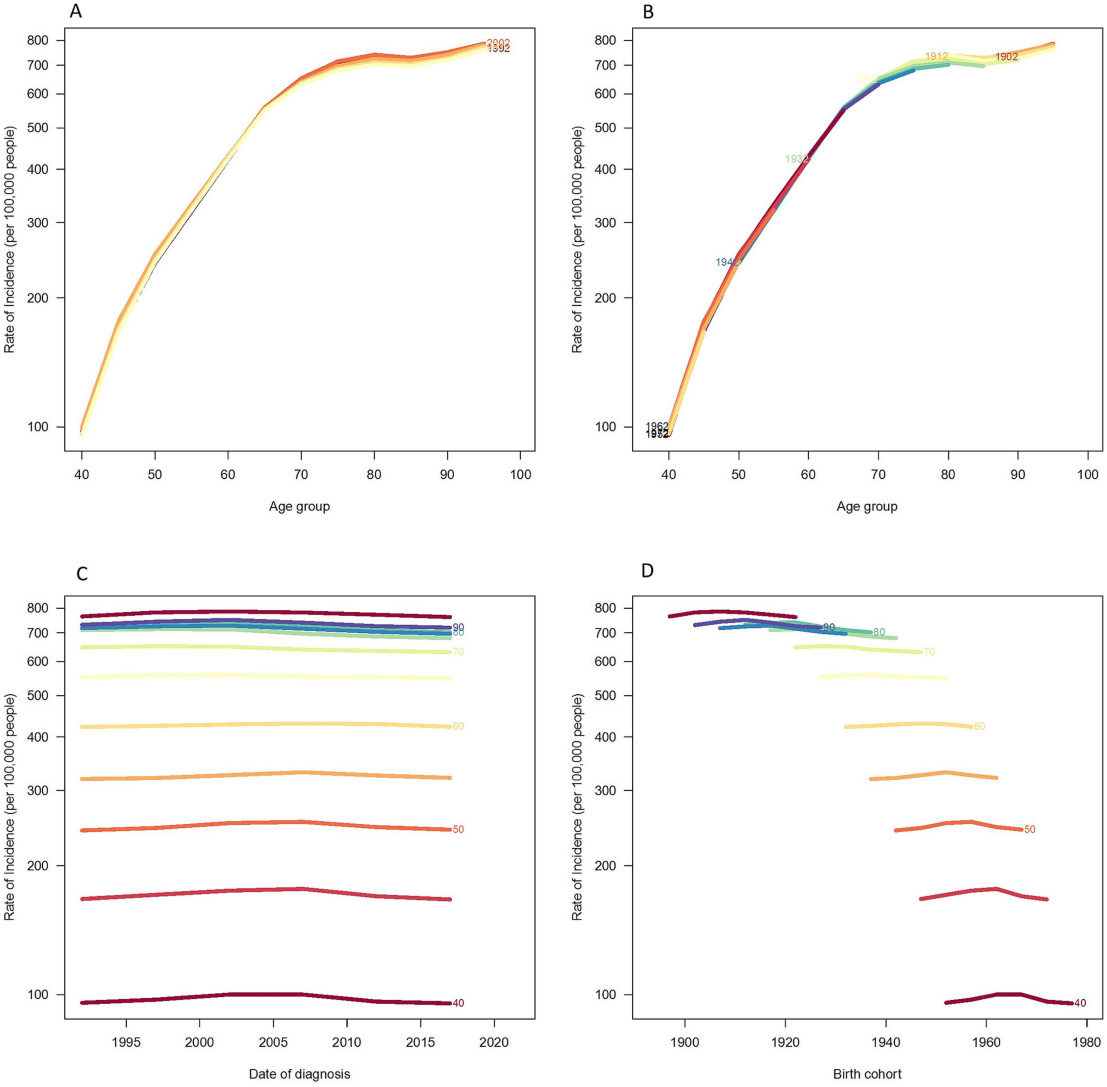
Incidence rates of PAD in China. **(A)** The age-specific incidences rates of PAD according to time periods; each line connects the age-specific incidence for a 5-year period. **(B)** The age-specific incidences rates of PAD according to birth cohort; each line connects the age-specific incidence for a 5-year cohort. **(C)** The period-specific incidence rates of PAD according to age groups; each line connects the birth cohort-specific incidence for a 5-year age group. **(D)** The birth cohort-specific incidence rates of PAD according to age groups; each line connects the birth cohort-specific incidence for a 5-year age group.

Figure 4A presents the trends in age-specific incidence rates during the years 1992, 1997, 2002, 2007, 2012, and 2017. The incidence rate of disease increases rapidly from ages 40 to 80 and then shows a gradual decline thereafter. Figure 4B describes the cohort trends in the incidence of lower extremity PAD across different age groups. Figure 4C describes the trends in the incidence of lower extremity PAD across different age groups from 1992 to 2021. The incidence rate for all age groups shows a slight increasing and then decreasing trend over time. Moreover, the incidence rate is higher in the older adult. Figure 4D describes the changes in incidence rates based on specific age cohorts. The incidence rate of lower extremity PAD increases with age. However, no significant differences are observed after the age of 80. The variations in incidence rates by age, period, and cohort for males and females are shown in the Supplementary Figures 3, 4.

**Figure 5:**
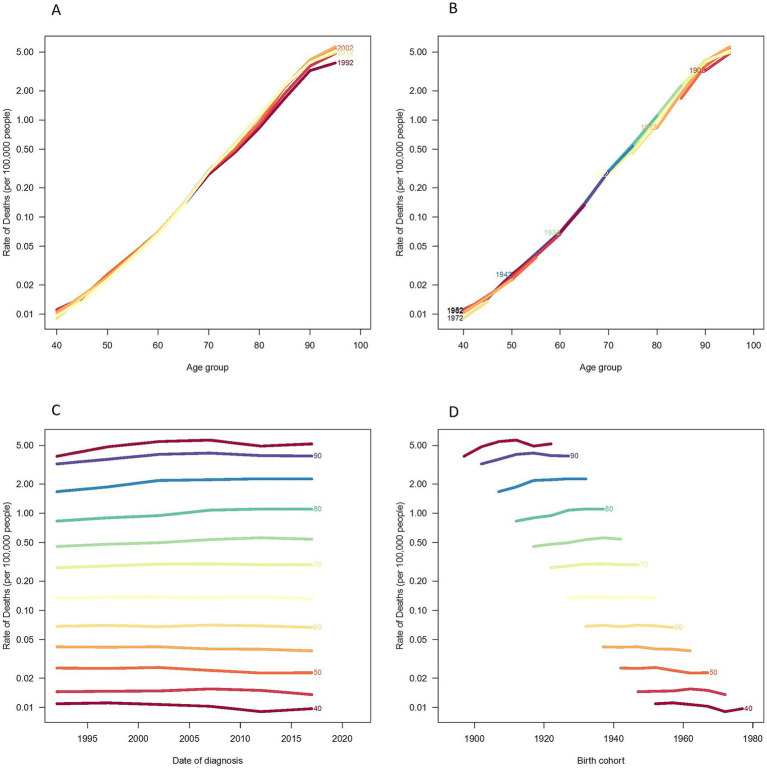
Mortality rates of PAD in China. **(A)** The age-specific mortality rates of PAD according to time periods; each line connects the age-specific mortality for a 5-year period. **(B)** The age-specific mortality rates of PAD according to birth cohorts; each line connects the age-specific mortality for a 5-year cohort. **(C)** The period-specific mortality rates of PAD according to age group; each line connects the birth cohort-specific mortality for a 5-year age group. **(D)** The birth cohort-specific mortality rates of PAD according to age groups; each line connects the birth cohort-specific mortality for a 5-year age group.

Figure 5A displays the trends in age-specific mortality rates during the periods of 1992, 1997, 2002, 2007, 2012, and 2017. Mortality rates increase rapidly from age 40 to 90, and although they continue to rise after age 90, the pace slows down. Figure 5B describes the cohort trends in mortality rates of lower extremity PAD across different age groups. Figure 5C describes the trends in mortality rates of lower extremity PAD across different age groups from 1992 to 2021. Moreover, the incidence rate is higher in the older adult. Figure 5D describes the changes in mortality rates based on specific age cohorts. The mortality rate of lower extremity PAD increases with age. The variations in mortality rates by age, period, and cohort for males and females are presented in the Supplementary Figures 5, 6.

**Figure 6:**
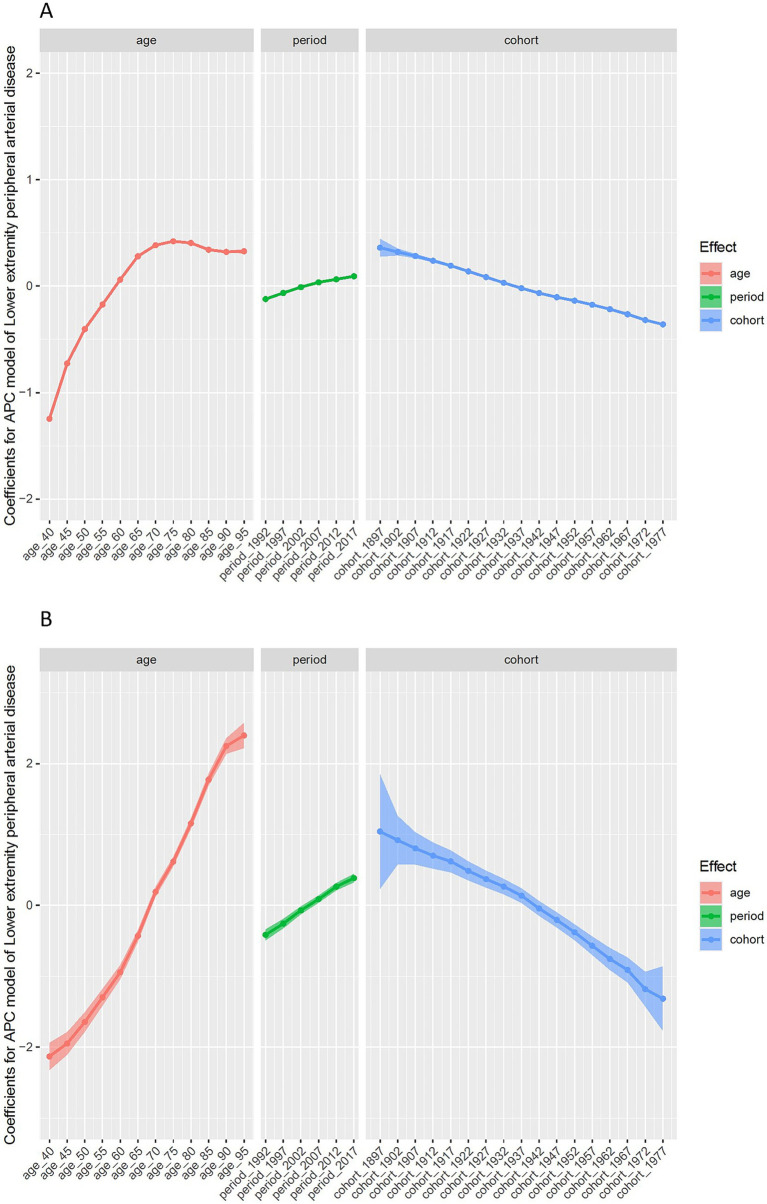
Estimated age-period-cohort effects for incidence **(A)** and mortality **(B)** of PAD in China (1992–2021).

Figure 6 illustrates the effects of age, period, and birth cohort on the incidence of lower extremity PAD (as shown in Figure 6A and Supplementary Table 3) and mortality rates (as shown in Figure 6B and Supplementary Table 4) to facilitate comparison among these effects. The age effect on the incidence of lower extremity PAD shows an increasing trend up to age 75, and then shows a decreasing trend after age 75, indicating that the risk of onset continues to rise with age up to 75, but slightly declines thereafter. The period effect indicates a continuous increase in the risk of onset with each passing year. The cohort effect suggests that later birth cohorts have a lower risk of onset compared to earlier ones. For mortality rates, the age effect indicates a continuous increase in risk with age, and the period effect shows a continuous increase in mortality risk over the years. The cohort effect indicates that later birth cohorts have a lower mortality risk compared to earlier ones.

### 3.4 ASIR and ASMR prediction of lower extremity peripheral arterial disease in men and women at 15 years

**Figure 7:**
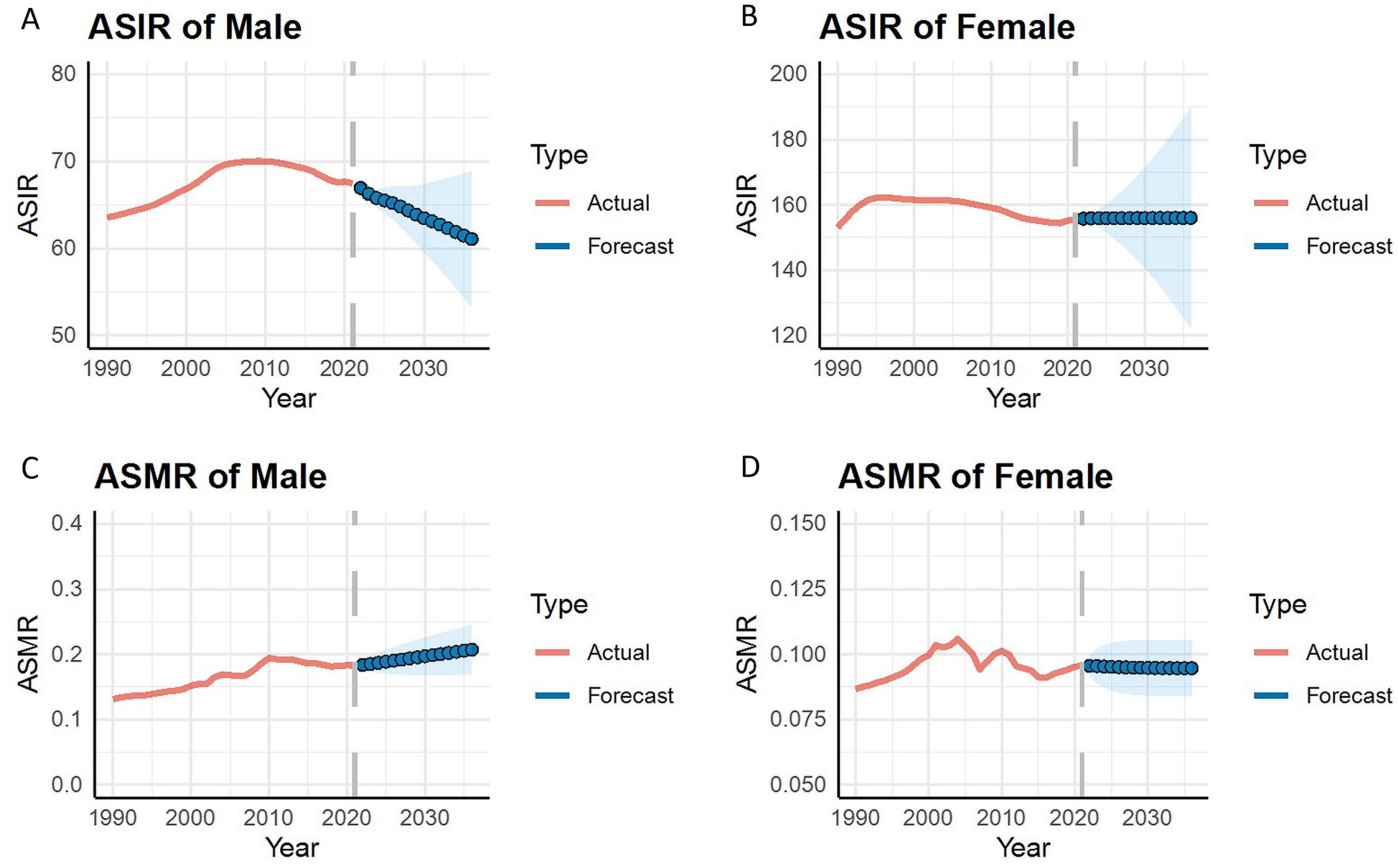
ARIMA model projections of age-standardized incidence rate **(A,B)** and age-standardized mortality rate **(C,D)** for the next 15 years from 2021.

As shown in Figure 7, future 15-year trend predictions were made for both male and female ASIR (A, B) and ASMR (C, D). In males, the optimal model chosen for ASIR is ARIMA (2, 2, 0) with AICc = −45.34 and RSME = 0.097. The optimal model chosen for ASMR is ARIMA (0, 1, 1) with drift, with AICc = −267.36 and RSME = 0.003. In females, the optimal model chosen for ASIR is ARIMA (1, 2, 0) with AICc = 14.65 and RSME = 0.279. The optimal model chosen for ASMR is ARIMA (2, 0, 0) with non-zero mean, with AICc = −295.72 and RSME = 0.002. All indicate that the model fits are relatively good and the predictive performance is quite accurate. The results suggest that the ASIR for lower extremity PAD in Chinese males will continue to decline over the next 15 years, while females will show a more stable trend. Surprisingly, the ASMR for males is expected to continue increasing over the next 15 years, while females will show a slight decrease.

## 4 Discussion

This study examines the trends in the burden of lower extremity PAD in China over the past 30 years and uses the ARIMA model to predict the ASIR and ASMR for males and females over the next 15 years. To our knowledge, this is the first epidemiological analysis to use the 2021 GBD database for lower extremity PAD in China with Joinpoint regression combined with the APC_IE model and ARIMA forecasting model. According to the previous 2019 GBD database, Chen et al. (14) published an epidemiological analysis of the global burden of atherosclerotic diseases, which only included partial results of the annual average change rate analysis of lower extremity PAD in China, differing from our results. Previous studies have found that some data differ with updates to the GBD database (15). However, insufficient analysis and differing data can lead to serious misunderstandings about the trends in the burden of lower extremity PAD in China among researchers. Therefore, conducting an in-depth analysis with the most recent data is particularly crucial.

Joinpoint regression analysis revealed that the increase in age-standardized prevalence rates from 1990 to 2021 was partly due to the decline in DALYs and YLDs, coupled with an increase in age-standardized incidence, mortality rates, and YLLs. The prevalence and incidence rates experienced a turning point around 2005, showing a declining trend, which may be influenced by advancements in medical technology and disease understanding (16, 17). This has effectively controlled the number of new cases and prevalent cases. However, the overall burden of lower extremity PAD in China still shows an increasing trend, which may be attributed to the rapid economic development and rapid changes in the social environment in China (18). Previous studies indicate that despite similar treatment patterns for PAD in China and Western countries, the awareness, treatment, and control rates of PAD among the general population in China are significantly lower. Data shows that only 4.9% of PAD patients in China are aware of their condition, only 1.9% have undergone revascularization, and only 0.2% of individuals achieved an ankle-brachial index greater than 0.9 (19). According to data from the Chinese National Clinical Research Center for Geriatric Diseases, the average hospitalization cost per PAD patient from 2008 to 2018 was 76,469.40 RMB (20). Although this is similar to the United States (21), China, as the world’s largest developing country, still faces a series of challenges such as unbalanced and insufficient regional development and large income disparities among residents. Therefore, as the world’s largest lower-middle-income country, the disease burden brought by lower extremity PAD in China remains relatively heavy (22).

Age effect is identified as a risk factor for lower extremity PAD in the constructed APC model. As shown in our results, the disease is common in people over the age of 40 (4). The results also indicate that among the age group of 40 to 95, females have higher prevalence and incidence rates than males, and mortality rates also show gender differences. This is consistent with previous research observations (23). The prevalence and incidence rates peak in the age group of 65–74, while mortality rates surge in the age group of 80–90, which is in line with global trends (4, 24). Relevant studies estimate that by 2050, China will have 400 million residents over the age of 65 (25). With the aging of the Chinese population, the high mortality and high YLLs rates among existing older adult PAD patients will likely alter the Chinese population structure or reduce life expectancy (26). An increase in the older adult population will lead to an increase in the number of patients, posing a significant challenge to China’s future healthcare system. PAD can cause significant trauma and disability, and it is estimated to cause $84 to $380 billion in losses to the U. S. economy annually (22). China may also face similar economic losses, which will not only hinder the modernization process of China’s economy but may also exacerbate regional economic imbalances (27, 28). Therefore, the prevention and control of PAD is not only related to public health but also an important issue in achieving sustainable socio-economic development.

Period effects are often influenced by a range of complex historical and environmental factors, such as war, economic crises, infectious disease epidemics, public health interventions, and socio-economic development. Although relevant studies have identified risk factors associated with the development of lower extremity PAD, such as tobacco consumption (4, 29), rapid industrialization has created an economic miracle for China but has also led to certain environmental pollution, posing new challenges to public health (30). Studies have shown that elevated levels of certain metals in urine are associated with a higher risk of PAD and have provided possible biological explanations (31). In addition, the increase in global plastic production and consumption has led to environmental pollution issues, including microplastics (32, 33). Studies have confirmed that bisphenol A (BPA), one of the chemical raw materials for plastic products, is significantly associated with the onset of PAD in urine, and this association is independent of traditional cardiovascular disease (CVD) risk factors (34). In addition, it has been found that plastic particles are not only present in human blood but also have a certain correlation with atherosclerosis (35). Although there is still relatively little research in this area, it provides a reasonable explanation for our research findings. Faced with the environmental degradation caused by rapid economic development, the Chinese government has clearly proposed strategic guidelines centered on sustainable development and high-quality growth (36, 37). This policy orientation aims to optimize industrial structures, promote green technological innovation, strengthen environmental regulation, and other measures to achieve coordinated development of economic growth and environmental protection. It is expected that the implementation of these policies will have a positive impact on improving environmental conditions in the future, promoting the long-term stability and healthy development of socio-economic conditions.

Cohort effects refer to the significant differences that may emerge among groups born in the same or similar time periods due to various social and environmental factors experienced throughout an individual’s life from birth to old age. This phenomenon indicates that members of different birth cohorts may exhibit different characteristics and trends in physiology, psychology, and social behavior due to their specific historical period backgrounds. Therefore, cohort effects emphasize the importance of considering an individual’s birth time and era background when studying population health, social changes, and behavioral patterns (38, 39). The study results show that cohorts born more recently have a lower risk of disease and death compared to those born earlier. Possible explanations for the differences among individual groups revealed by cohort effects are multifaceted. First, advancements and innovations in medical technology may significantly impact population health (40). Second, improvements in dietary conditions and changes in nutrition intake may also affect the physiological health of different birth cohorts (41). Furthermore, adjustments in national policies, such as those in healthcare, may also differentially affect individuals from different cohorts (42). Lastly, changes in lifestyle, including work patterns, leisure activities, and social habits, may also significantly differ among cohorts (43). These factors collectively shape the unique characteristics of different cohort members, thus creating observable differences among groups.

The ARIMA forecast analysis indicates that over the 15 years from 2021 to 2036, the ASIR and ASDR for lower extremity PAD in Chinese females are expected to remain stable, but no significant decline is anticipated. For Chinese males, the ASIR for lower extremity PAD is expected to decrease, while the ASDR shows a gradual increasing trend. This forecast suggests that the focus on and intervention needs for male PAD patients may further increase in the future.

The study has several limitations. Firstly, the estimated data based on complex statistical models may have certain uncertainties and inaccuracies. Secondly, the predictions of this study are limited to the burden trends of ASIR and ASDR and do not cover other aspects of the PAD disease burden, such as disability-adjusted life years (DALYs). Furthermore, due to China’s vast territory and diverse ethnic groups, there may be significant epidemiological differences between provinces and ethnic groups, but the data available to assess these differences is relatively limited, which may affect the generalizability of the study’s results to some extent.

Despite these limitations, this study utilizes the latest Global Burden of Disease (GBD) data, combined with advanced statistical models, to provide valuable forecasts on the future trends of lower extremity PAD disease burden in China. These forecast results can provide a scientific basis for public health decision-makers and healthcare providers to develop relevant policies and intervention measures, particularly in focusing on and intervening with male PAD patients, in order to mitigate the impact of PAD on China’s socio-economic and public health.

## 5 Conclusion

This epidemiological analysis is based on the latest 2021 Global Burden of Disease (GBD) data, revealing a significant increase in the number of lower extremity PAD patients in China over the past three decades. The study employs rigorous statistical modeling and forecasting techniques, indicating that PAD poses a significant public health challenge to the healthcare system, with significant impacts on both disability and mortality rates. Despite advancements in medical technology and increased public awareness, PAD remains a significant burden on the healthcare system. Future disease burden trend forecasts show a continued increase, which could place immense pressure on healthcare resources. Therefore, there is an urgent need for a multifaceted intervention approach, including clinical treatment strategies and public health measures such as lifestyle changes, risk factor control, and public education programs, to alleviate the disease burden of PAD. The forecasted trend for the next 15 years indicates that without effective measures, the disease burden of PAD may further increase, placing greater pressure on healthcare resources. Therefore, there is an urgent need for a comprehensive approach to managing PAD, including medical policy and community engagement, to effectively address this growing public health issue.

## Data Availability

The original contributions presented in the study are included in the article/Supplementary material, further inquiries can be directed to the corresponding author.

## Footnotes

^1^ https://vizhub.healthdata.org/gbd-results/

^2^ https://github.com/robjhyndman/forecast
