# Supplementary figures and images for: A trend analysis of the burden of lower extremity peripheral arterial disease in China, 1990 to 2021: based on the Global Burden of Disease Study 2021
Source: Front Public Health. 2025 Jun 19;13:1506748. doi: 10.3389/fpubh.2025.1506748 (PMC12221934; doi:10.3389/fpubh.2025.1506748)

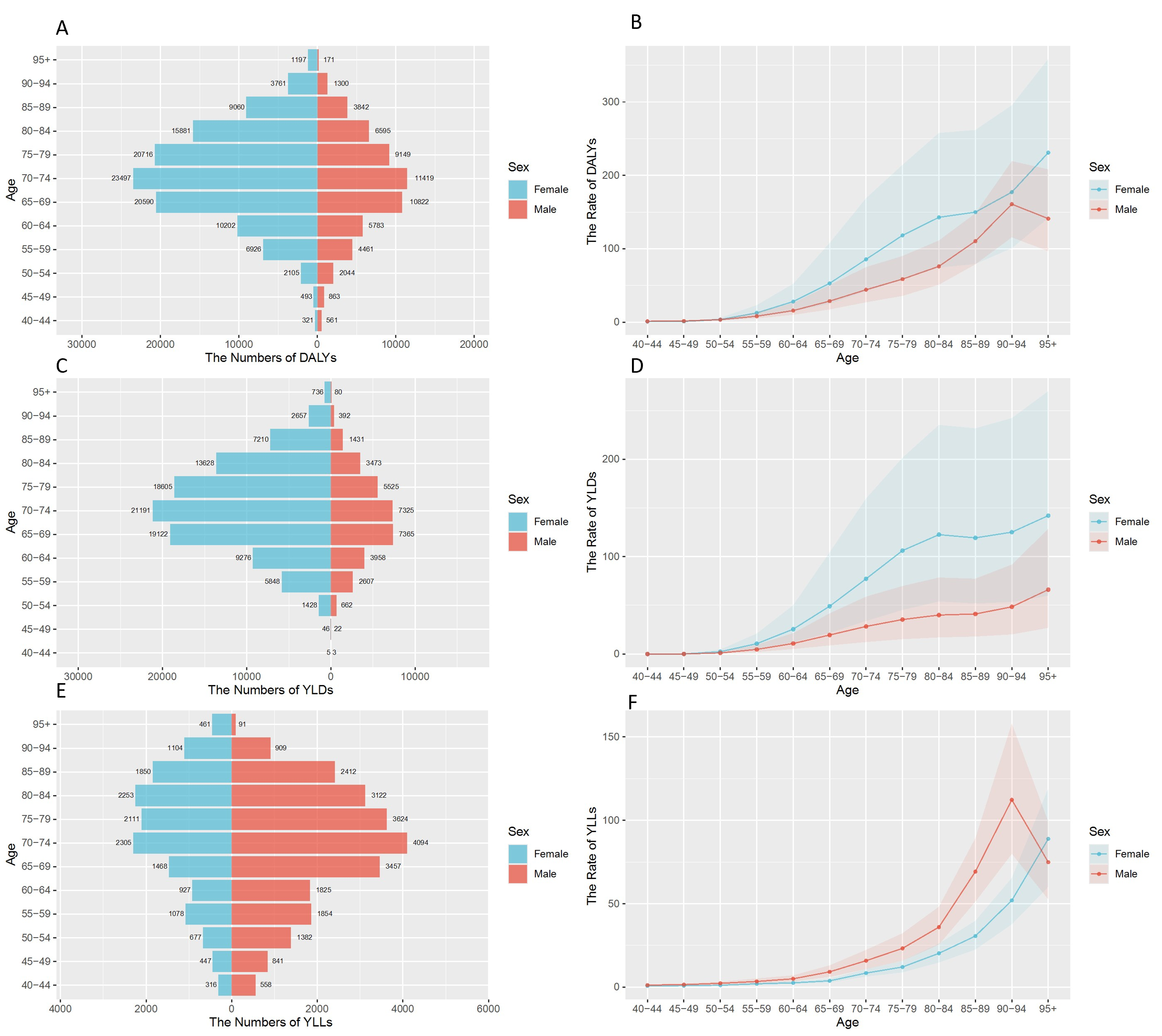

Supplement: Supplementary file 1 [file Data_Sheet_1.zip › Supplementary materials/Figure 1 (Supllementary).tif]

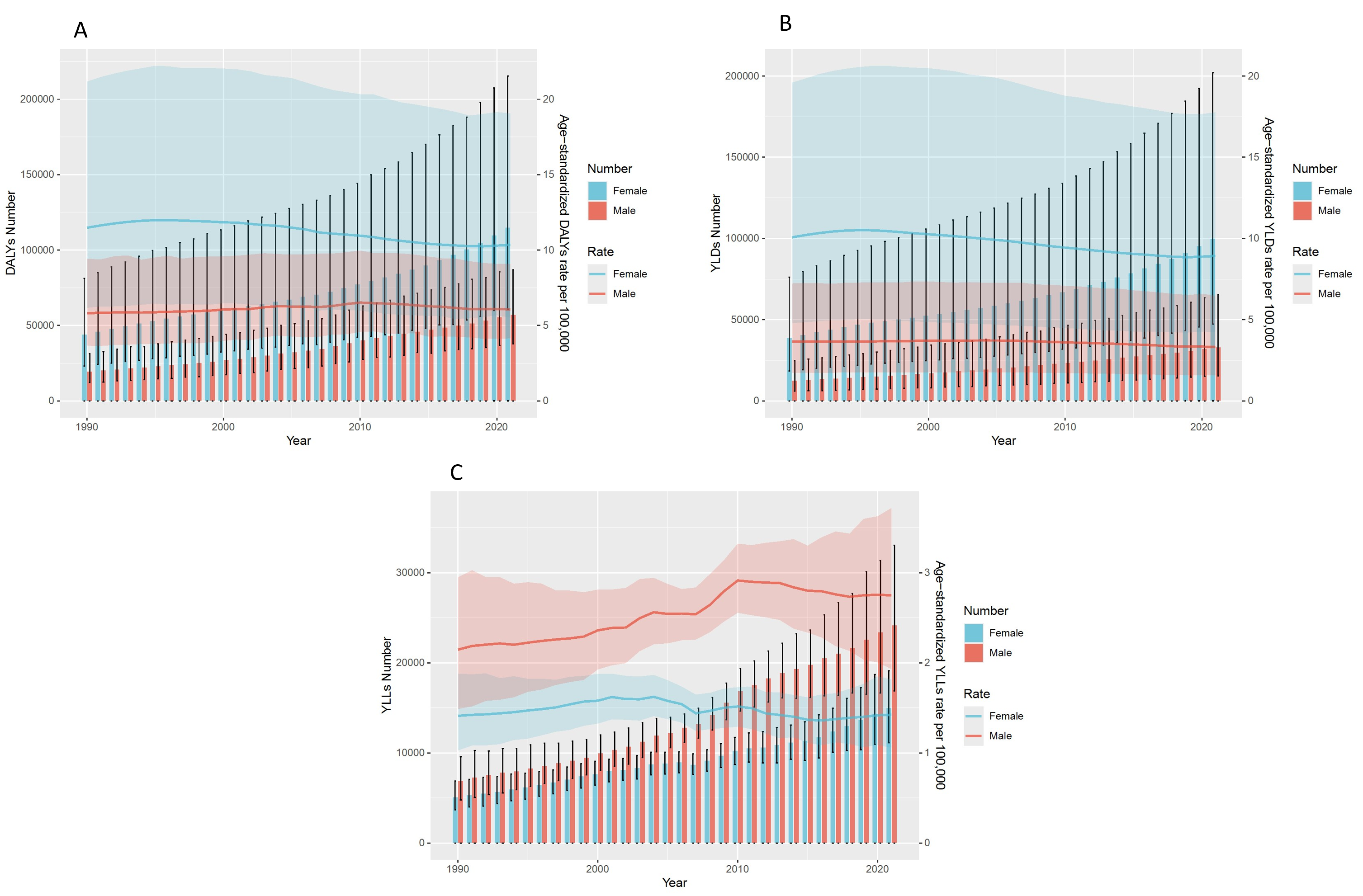

Supplement: Supplementary file 1 [file Data_Sheet_1.zip › Supplementary materials/Figure 2 (Supplemetary).tif]

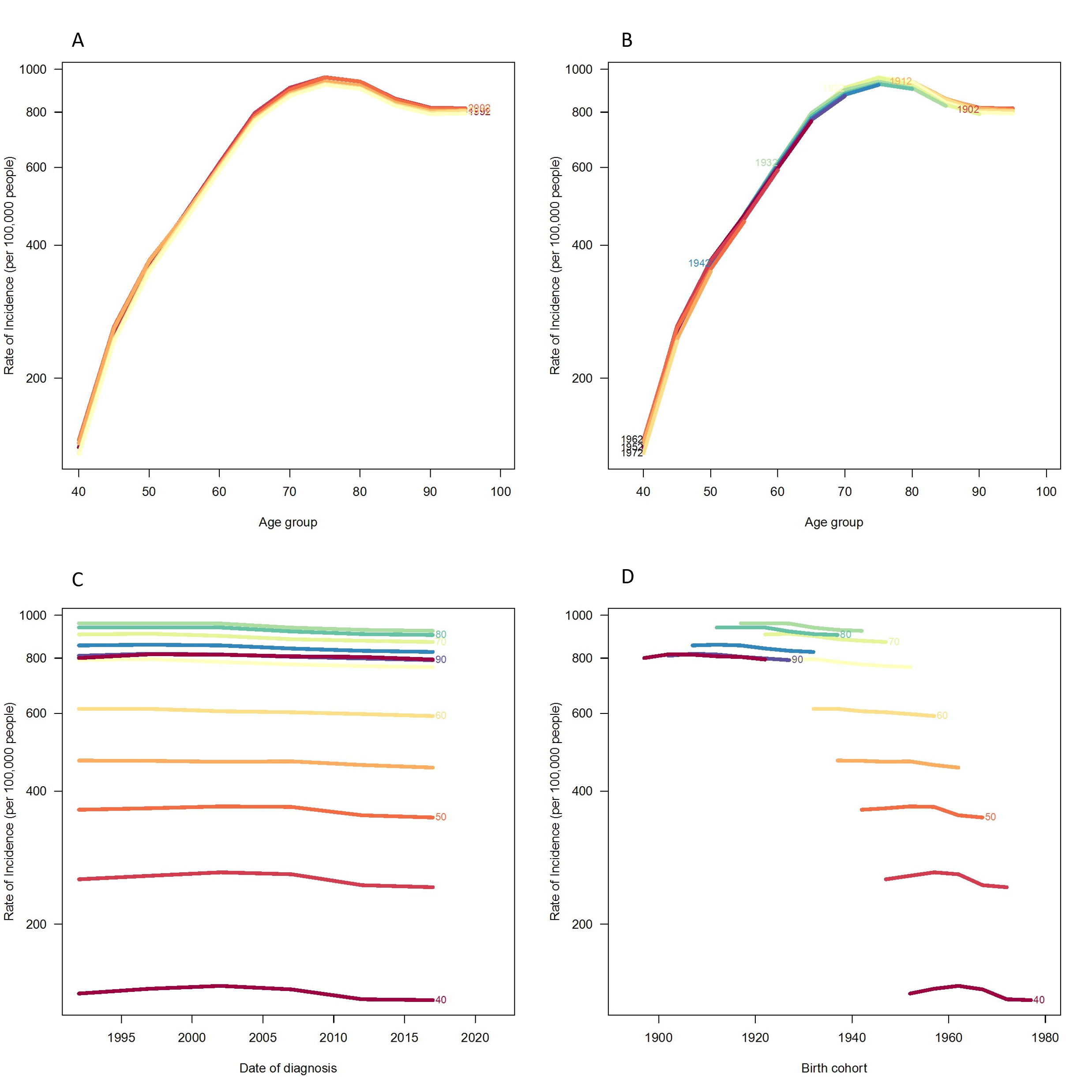

Supplement: Supplementary file 1 [file Data_Sheet_1.zip › Supplementary materials/Figure 3(Supplemetary).tif]

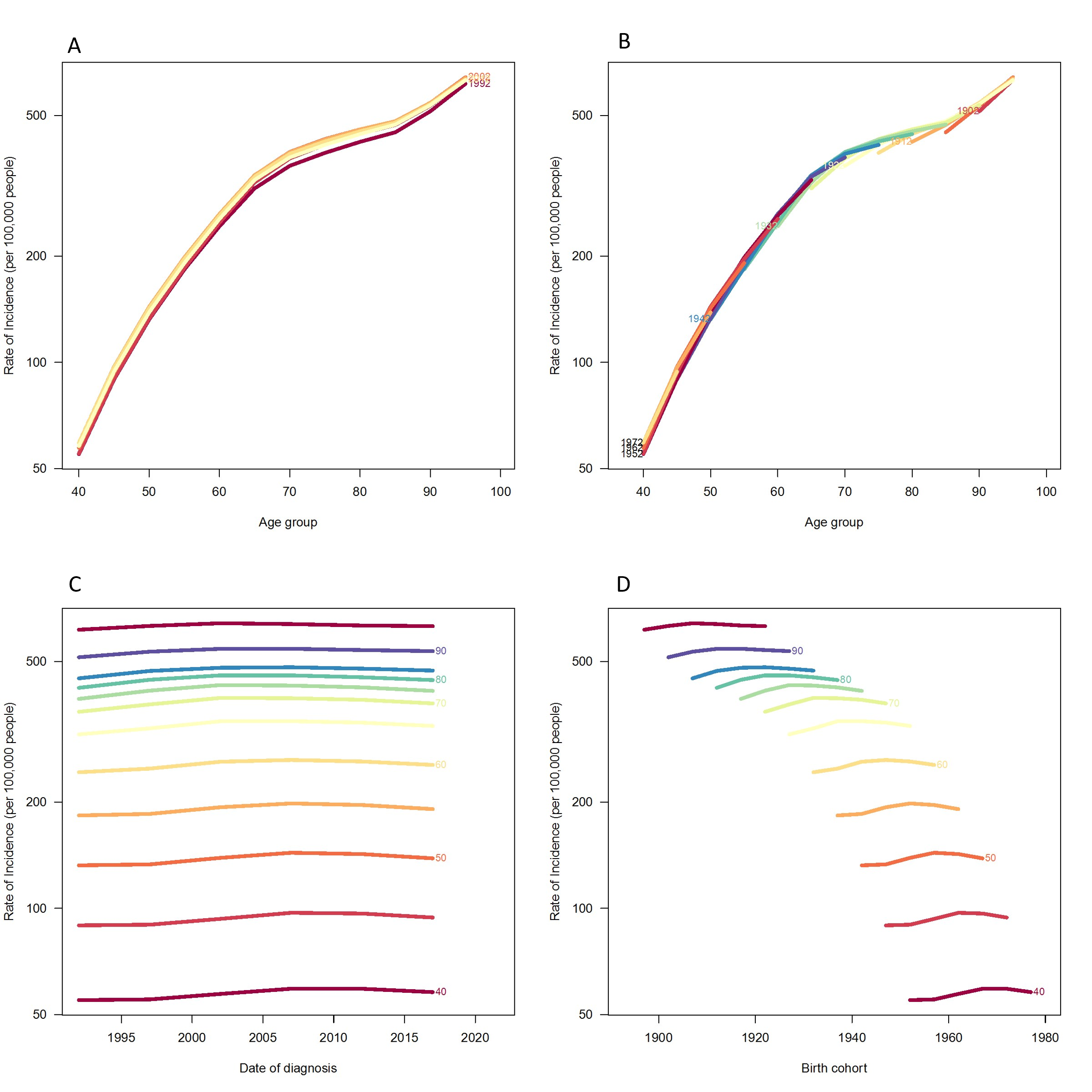

Supplement: Supplementary file 1 [file Data_Sheet_1.zip › Supplementary materials/Figure 4 (Supplemetary).tif]

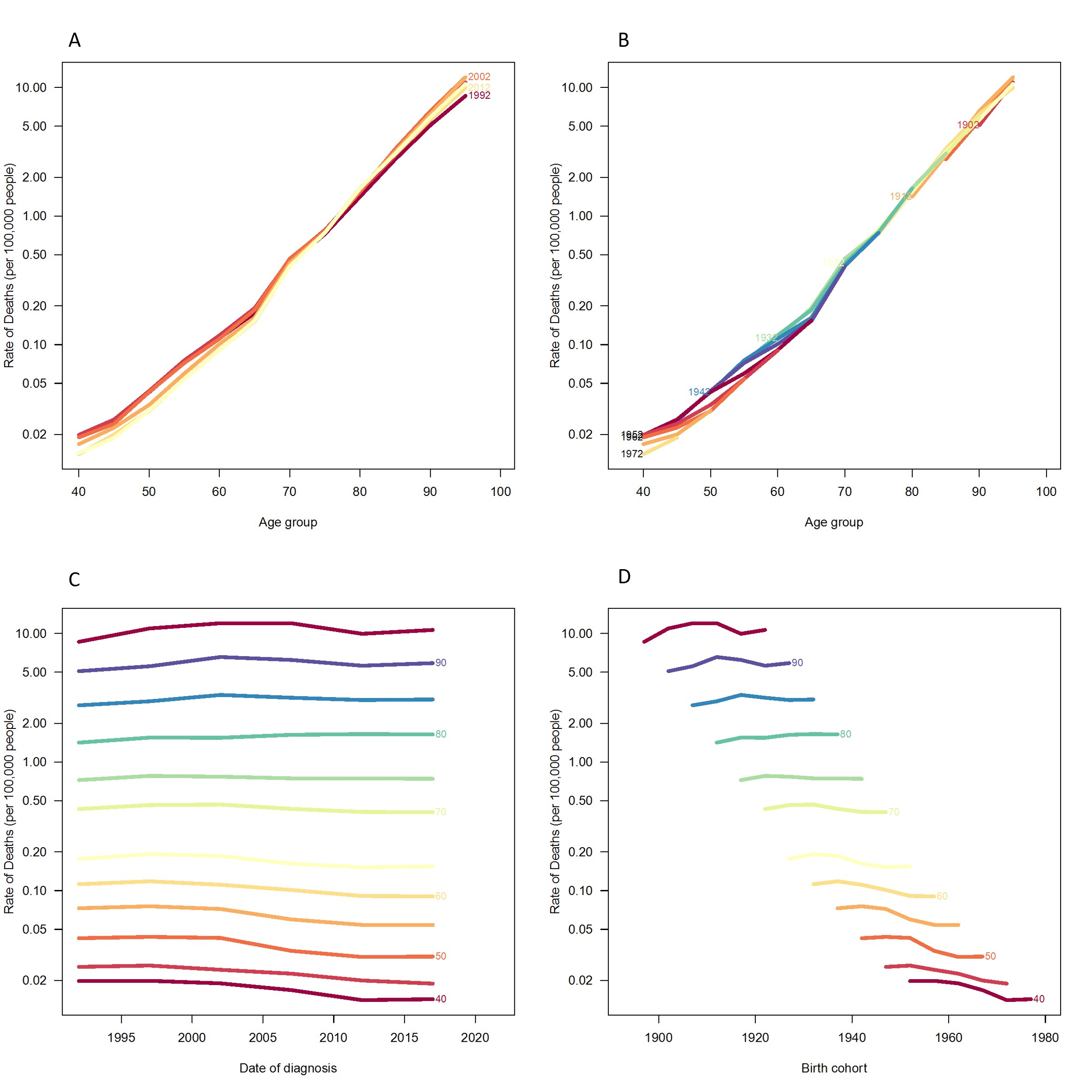

Supplement: Supplementary file 1 [file Data_Sheet_1.zip › Supplementary materials/Figure 5 (Supplemetary).tif]

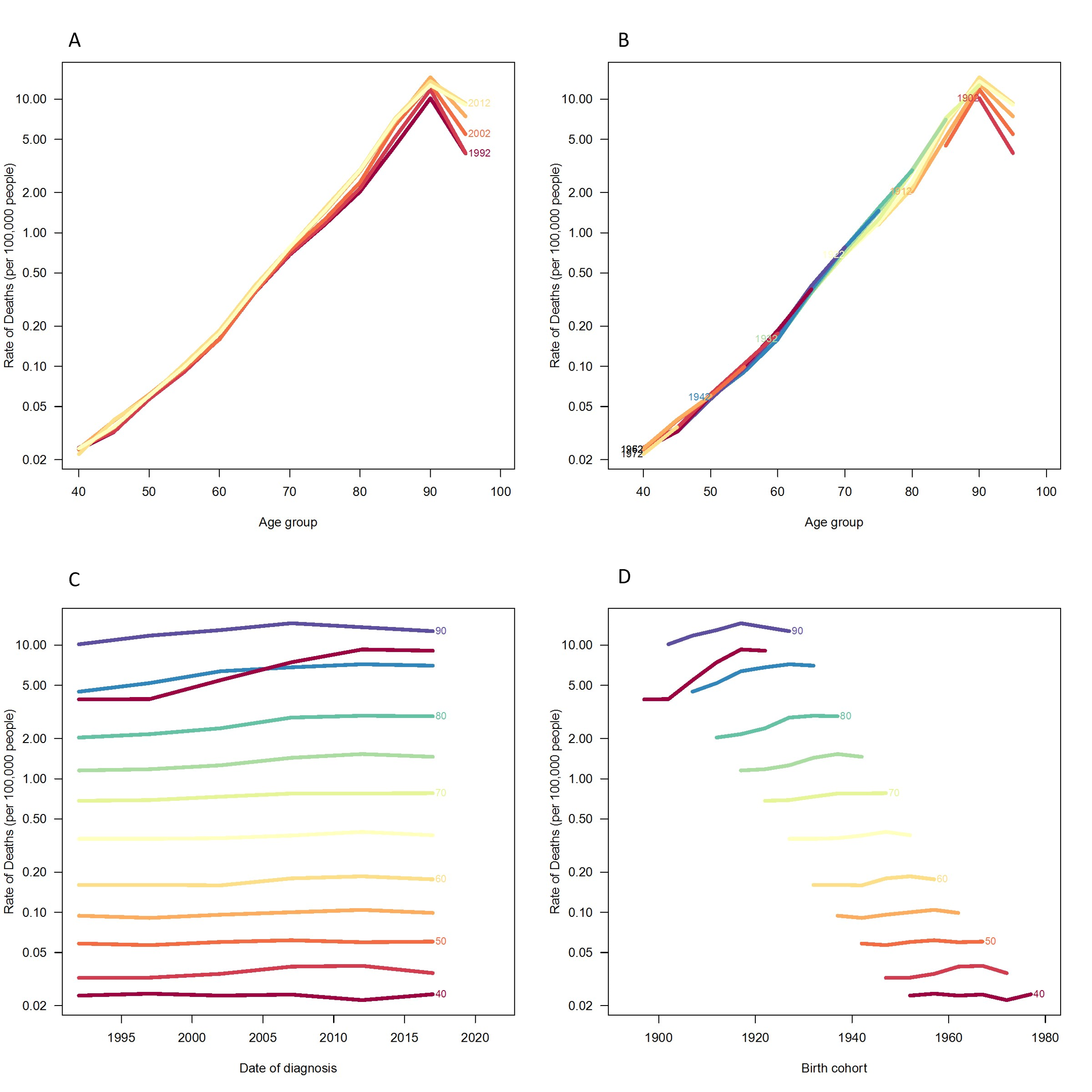

Supplement: Supplementary file 1 [file Data_Sheet_1.zip › Supplementary materials/Figure 6 (Supplemetary).tif]
